# Modeling Concurrent Hedonic Disruption During Excessive Drinking and Abstinence‐Related Negative Affect in a Novel Selectively Bred Mouse Line: The Crossed High Alcohol Preferring X High Drinking in the Dark (cHAPxHDID) Mice

**DOI:** 10.1111/acer.70400

**Published:** 2026-08-20

**Authors:** Garrett A. Winkler, Nicholas J. Grahame

**Affiliations:** ^1^ Department of Psychology Indiana University Indianapolis Indianapolis Indiana USA

**Keywords:** alcohol abstinence, anhedonia, comorbidity, depression, selective breeding

## Abstract

**Background:**

Alcohol use disorder (AUD) is often comorbid with depressive disorders such as major depressive disorder (MDD). These comorbidities are often modeled by allowing C57BL/6 (B6) mice to drink alcohol and testing for depressive‐ and anxiety‐like phenotypes 2–3 weeks into abstinence; however, it should be noted that B6 mice are both inbred, which may limit external validity, and show modest intakes compared to humans with AUD. We have created a new selectively bred line of high alcohol‐preferring mice, the crossed High Alcohol Preferring X High Drinking‐in‐the‐Dark mice (cHAPxHDIDs). The cHAPxHDID mice have not been explored as a model of AUD + MDD comorbidity, nor has their drinking propensity been characterized in any publication to date.

**Methods:**

We used male and female cHAPxHDID mice and a home cage 2‐bottle choice (2BC) paradigm (water/water v. 10% ethanol/water) for 5 weeks across three experiments. We characterized the cHAPxHDIDs' 2BC drinking by measuring intake over a day, along with blood ethanol concentrations (BECs). Additionally, we ran behavioral tests for negative affect at different timepoints in abstinence. Finally, we also probed for the emergence and sustenance of anhedonia during the drinking period with a modified 1% sucrose preference test (SPT).

**Results:**

We found that cHAPxHDID mice drink excessively, especially at the onset of the dark cycle, and achieve average BECs higher than 300 mg/dL. Ethanol drinkers showed increased latency in the novelty‐suppressed feeding test (NSFT) at 6 days into abstinence. Alcohol‐drinking mice reduced their sucrose intake during the drinking period beginning in Week 2, which normalizes at 48 h into abstinence.

**Conclusions:**

The cHAPxHDIDs show some evidence of depressive symptomatology after and during drinking. They drink excessive amounts of alcohol voluntarily, reaching high and sustained BECs. Taken together, our data suggest that the cHAPxHDIDs may be a promising model for excessive drinking and its mood‐related comorbidities.

## Graphical abstract

A new line of selectively bred mice, cHAPxHDID, shows high alcohol intake and blood alcohol levels that cause anhedonia during alcohol consumption. This quickly resolves in abstinence, and behavior indicative of depression emerges 6 d into abstinence.

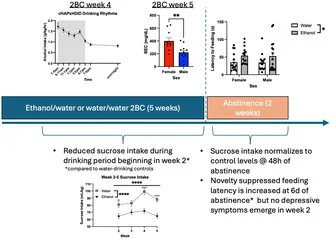

## 1 Introduction

Alcohol use disorder (AUD) is characterized by excessive alcohol drinking and a preoccupation with obtaining and consuming alcohol, often in the face of negative life consequences and health risks (American Psychiatric Association 2013). In addition to these criteria, symptoms likely also include tolerance, whereby individuals require more alcohol to acquire the desired level of intoxication, and withdrawal‐related symptoms such as tremors, headaches, or psychological symptoms like anxiety and depressed mood (Becker 2008). Alcohol consumption, sensitivity, tolerance, and withdrawal have been extensively studied using mouse models in an attempt to recapitulate AUD and uncover traits, behaviors, circuits, and molecular markers associated with heavy drinking and alcohol preference (Becker 2000; Becker and Ron 2014; Egervari et al. 2021; Matson et al. 2014; Perez and De Biasi 2015). The most widely used of these models is 2‐bottle choice home cage drinking (2BC), whereby animals have continuous access to a choice between 10% (v/v) ethanol and water (Huynh et al. 2019; Tabakoff and Hoffman 2000). The 2BC method is simple, naturalistic, and voluntary, providing face validity to this model.

The inbred mouse strain most often used in 2BC, C57BL/6 (B6) mice, strongly prefer alcohol over water, often reaching > 80% preference after 3–4 weeks of drinking, with some studies reporting intakes of ~20 g/kg/24 h or higher (Hwa et al. 2011; Yoneyama et al. 2008). While this is on the high end of inbred strain intakes, these mice often do not reach sustained intoxicating blood ethanol concentrations (BECs) using 2BC. This contrasts with the prolonged high BECs seen in human AUD patients (Dole and Gentry 1984; Hämäläinen et al. 2018), some of whom may engage in high‐intensity drinking where a person regularly drinks two to three times the binge drinking threshold of 80 mg/dL (Patrick and Azar 2018). In previous publications, our lab has demonstrated the disparity in BEC and alcohol intake during 2BC between B6 mice and the lines of selectively bred High Alcohol Preferring (HAP) mice (Matson and Grahame 2013). In short, all HAP lines show high intakes and moderate to high BECs, but the HAP1 line and the crossed High Alcohol Preferring line (cHAP; a cross between HAP1 and HAP2 replicates) show BECs that average well above 200 mg/dL. B6 mice, in comparison, show average BECs around 60 mg/dL (Matson et al. 2013; Matson and Grahame 2013). HAP lines, especially the cHAPs, with their high and persistent intakes and BECs, may be of translational relevance to the study of high‐intensity alcohol consumption, its predictors, its consequences, and its relevant comorbidities.

One such AUD comorbidity is major depressive disorder (MDD) and other related mood disorders. As discussed in our recent translational review of MDD + AUD comorbidities (Winkler and Grahame 2025), depressive symptoms can predate an AUD diagnosis, coincide with one, or may develop following drinking. In the majority of those individuals with symptoms of depression in alcohol withdrawal and into abstinence, mood‐related disturbances tend to wane at 2–4 weeks post‐cessation (Brown and Schuckit 1988; Foulds et al. 2015; Liappas et al. 2002; Rabinowitz et al. 2023). This is in stark contrast to the preclinical fields' investigations of alcohol abstinence‐related correlates of depressive‐ and anxiety‐like behaviors in mice (Holleran and Winder 2017), specifically B6 mice: these animals seem to exhibit an anxiety‐like phenotype in early abstinence (12–48 h) and a depressive‐like phenotype in protracted abstinence (2–3 weeks), especially following a continuous 2BC procedure (Bloch et al. 2022). So, in this strain of mice, depressive behaviors peak at the same time they are abating for most human AUD patients who present with post‐quit mood‐related symptoms. Previously, we have explored these behaviors in cHAP mice using the same 2BC drinking history and similar abstinence periods, but we found an increase in sucrose preference in the sucrose preference test (SPT) for anhedonia after 1 week of abstinence and no differences between alcohol or water history in animals in the forced swim test (FST) or the novelty suppressed feeding test (NSFT) at either 1 or 3 weeks of abstinence (Winkler and Grahame 2024). Normally a decrease in sucrose preference is taken as evidence for anhedonia, a lack of pleasure derived from endogenously or previously pleasurable stimuli (Gencturk and Unal 2024; Liu et al. 2018), so this seeming increase in pleasure from sucrose following 2BC and abstinence compared to water controls is difficult to interpret, though this has been observed in B6 as well (Lee et al. 2017).

As stated, though depressive symptoms following cessation from drinking do tend to dissipate during abstinence, anhedonia in early abstinence is a symptom that correlates with future prognosis of both AUD and other depressive symptoms in late abstinence (Destoop et al. 2019; Garfield et al. 2014; Nguyen et al. 2020; Petit et al. 2020). Again, anhedonia via the SPT is most commonly measured 2–3 weeks into abstinence (Bloch et al. 2022), a timepoint that may overshoot peak anhedonia in humans. This early onset anhedonia in humans may especially be prevalent in those patients experiencing concomitant AUD and depression (Ryan et al. 2022), whereby the anhedonia associated with repeated withdrawal could exacerbate future drinking and perpetuate the severity of both disorders. This type of comorbidity in “current drinkers” may be underexplored in preclinical models because, in contrast with clinical models where subjects can self‐report while intoxicated, animal models often rely on some locomotive component of behavior to determine whether an animal is exhibiting negative affect. Alcohol interactions with locomotion are, of course, a potential confound in testing animals that are actually currently intoxicated, not to mention that many of these tests for depression and anxiety cannot be repeated, adding another confound to longitudinal testing (Winkler and Grahame 2025). Therefore, in these experiments, we explore anhedonia and depressive‐like behaviors using a new selectively bred line of mice that drinks alcohol as intensely as other HAP lines called cHAPxHDID (see below), but has a somewhat different genetic background, to explore two parallel research questions. First whether, similar to humans, there is anhedonia in “current drinkers” (which here is operationalized as animals that could drink 12 h ago but which are currently sober), and second, whether there are symptoms of anhedonia and depression in animals that have abstinence periods longer than 12 h. To achieve the first goal, we used a modified SPT probe procedure that we repeated weekly during the chronic drinking period and/or at early and later timepoints into abstinence that reflect when an AUD patient would most likely report distressing anhedonic symptoms. To explore the second goal, we used more conventional testing for depressive‐like behaviors, including NSFT and FST, at abstinence timepoints of at least 48 h.

The crossed High Alcohol Preferring X High Drinking‐in‐the‐Dark mice (cHAPxHDID) are a selected line whose progenitor population was created from a cross between Dr. John Crabbe's High Drinking in the Dark (HDID) line (Barkley‐Levenson and Crabbe 2014), bred to drink large amounts of alcohol in Drinking‐in‐the‐Dark (DID—a binge drinking paradigm), and the cHAPs mentioned earlier (see Methods for more information about the generation of the cHAPxHDIDs). In the past few years and through a handful of experiments, our lab has attempted to understand how and why cHAPxHDID mice drink excessively in 2BC and DID procedures in order to determine if they might more flexibly model the varieties of human AUD and its comorbidities than commonly used inbred strains. Here, along with demonstrating the creation of this selected line over generations, we characterize two‐bottle choice drinking behavior and associated blood ethanol levels (BECs), as well as using the model to explore anhedonia and depression in both current drinkers and following more prolonged abstinence periods, though still short enough to be relevant to human timelines of abstinent‐related depression and anxiety symptoms. This latter study is based on suggestions from a recent review paper (Winkler and Grahame 2025) showing divergence between clinical and preclinical studies in affective changes during and after voluntary alcohol drinking. Based on their impressive daily intakes, we hypothesized that they would achieve levels of intoxication that might exceed those seen in cHAPs (Matson et al. 2013) and that their high‐drinking behavior would perhaps create conditions that more closely adhere to anhedonia and depression in humans with more severe drinking histories.

## 2 Materials and Methods

### 2.1 Animals

All experiments utilized cHAPxHDID animals, bred in IUI Animal Care Facilities. The progenitors for these selectively bred animals were derived by reciprocally crossing selectively bred High Drinking in the Dark 1x2 mice from Dr. John Crabbe at OHSU with crossed High Alcohol Preferring mice. In detail, S26 HDID1 mice were reciprocally crossed with S20 HDID2 mice to create an HDID1x2 line that became one of the two lines that were subsequently reciprocally crossed to create cHAPxHDID progenitors. The other line was S27 of the cHAP line (for more detail on derivation of cHAP mice, see Oberlin et al. (2011)). For the first four generations, there were two selection criteria: BEC following the fourth of four DID sessions and high g/kg intake during a subsequent 2‐week, 2BC access. Generally, there were 4 days of no alcohol access between DID and 2BC testing. After selection response for DID BECs proved modest (consistent with its considerably lower heritability than alcohol intake during 2BC), we elected to continue selection using 2 weeks of 2BC only and continue this selection procedure at the time of writing. Selection methods were the same as those used for the HAP lines (Oberlin et al. 2011): generally, mass selection was used, with the exception that first‐cousin matings were avoided to slow the rate of inbreeding. The only difference was the use of a shortened, 2‐week 2BC access period instead of the 4 weeks used for the HAP lines. The cHAPxHDID line has shown a robust response to selection for 2BC (see Figure 2), and parents of the mice used in this study drank an average of 29.33 g/kg per day during the 2‐week phenotyping period.

In Experiment 1, 42 alcohol‐naïve cHAPxHDIDs (21M, 21F) from generation 27 of selection were used, aged 94–106 days. In Experiment 2, 38 alcohol‐naïve cHAPxHDIDs (19M, 19F) from generation 28 were used, aged 98–104 days. In Experiment 3, 72 alcohol‐naïve cHAPxHDIDs (36M, 36F) from generation 31 were used, aged 101–104 days. Experiment 3 acted as a replication of both the post‐drinking testing in Experiment 1 and the during‐drinking testing in Experiment 2. All animals were single‐housed 1 week prior to alcohol access and had *ad libitum* access to water and chow when in the home cage. Cages were changed biweekly after single‐housing, and individual animal weights were recorded weekly. Mice were maintained on a reverse 12:12 light–dark cycle with lights off at 0700 in Experiments 1 and 2 and 0800 in Experiment 3. Before drinking began, animals were counterbalanced by sex and family to either water or ethanol groups. All procedures were IACUC‐approved and conducted at an AAALAC‐approved facility adhering to the National Research Council's Guide for the Care and Use of Laboratory Animals.

### 2.2 Free‐Choice Drinking

**FIGURE 1:**
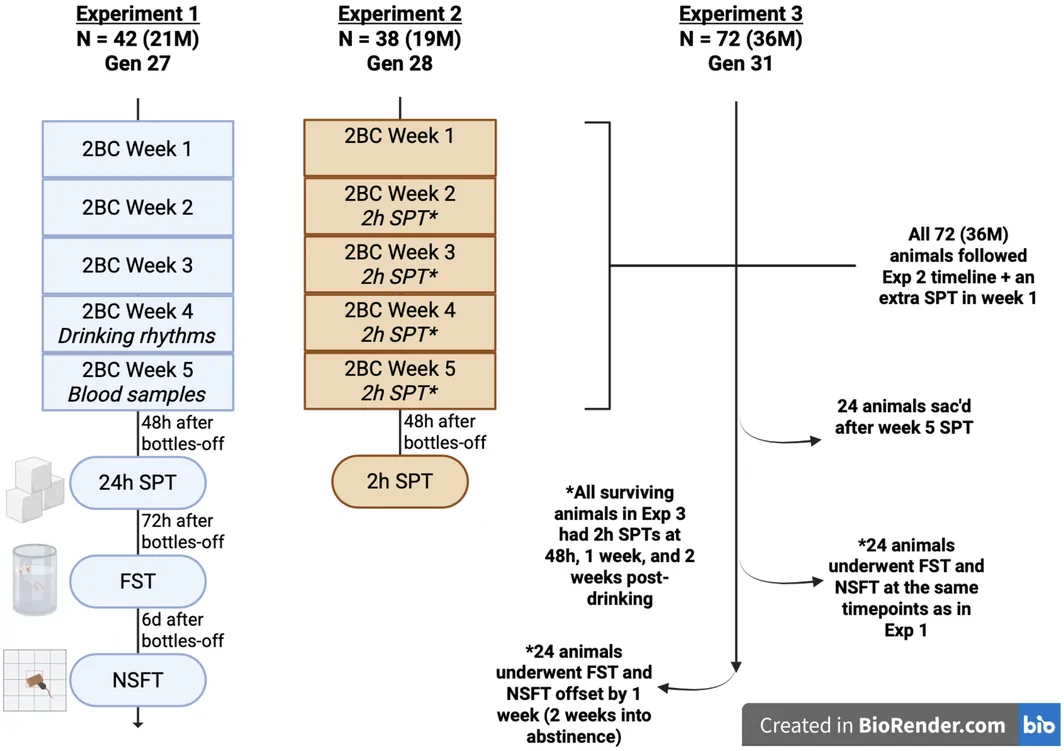
Experimental timelines. In Experiment 1, 42 mice underwent 5 weeks of 2BC followed by depression testing (SPT, FST, NSFT) in early abstinence. In Week 4, drinking rhythms were recorded and blood samples were taken in Week 5. In Experiment 2, 38 mice underwent the same 5‐week 2BC schedule. Starting in Week 2, animals had a 2‐h SPT* probe to check for anhedonia. Experiment 3 served as a replication of the anhedonia testing in Experiment 2, followed by the FST and NSFT in Experiment 1. A subset of 24 animals in Experiment 3 underwent FST and NSFT 2 weeks into abstinence to understand protracted phenotypes. In Experiment 3, all surviving animals had abstinence SPT at 48 h, 1 week, and 2 weeks into abstinence. *In Experiment 2, we used a modified sucrose preference test during the drinking period. Each week starting on Week 2, alcohol was removed from the cage at lights‐on and 12 h later at lights‐off, sucrose and water sippers were introduced for 2 h and then replaced with alcohol and water following the test. The final 2‐h test was run 48 h into abstinence.

In all experiments, after a week of single‐housing, water bottles were removed and replaced with either two 50 mL graduated cylinders of tap water (water controls) or one 50 mL cylinder of tap water and another 50 mL cylinder of 10% v/v ethanol in tap water, diluted from 200 proof ethanol stock (Pharmco Inc.; Brookfield, Connecticut, United States). On Mondays, Wednesdays, and Fridays, intakes were measured by reading the meniscus of the liquid in the cylinder on the cage to the closest 0.5 mL, and cylinder positions were swapped on these days to control for spatial preference. Cylinders remained on the cage for 5 weeks (35 days) except where explicitly mentioned and were replaced with a home cage water bottle at the conclusion of the drinking period. The experimental timeline for all experiments is displayed in Figure 1.

### 2.3 Experiment 1–24‐h Drinking Rhythm Assessment

At the end of the fourth week of drinking at lights‐off (0700), 50 mL cylinders were replaced with 10 mL serological pipette sippers readable to the nearest 0.05 mL. This swap occurred for ethanol‐drinking animals only and not water controls, and the relative positions of the solutions on the cage were conserved over the swap to these sippers. Every 2 h of the dark cycle, the volume of the sippers was recorded in order to track intake during a typical day of drinking for these animals. An additional reading was taken 2 h after lights‐on, and then the sippers were topped off and left on the cage for the next 10 h, with a final reading occurring at lights‐off the next day. Pipette sippers were replaced with the 50 mL graduated cylinders, swapping the position of the ethanol and water on the cage.

### 2.4 Experiment 1—Blood Ethanol Concentrations

Five days later, retro‐orbital blood samples were taken from all animals regardless of alcohol history using heparinized capillary tubes at 1400 (7 h into the dark cycle). Samples were stored on ice in heparinized centrifuge tubes until all samples were collected and subsequently centrifuged at 14,000 rpm at 4 C for 5 min. Plasma supernatant was separated and stored at −20 C until time of analysis. Only samples from ethanol‐drinking mice were analyzed via Analox EtOH Analyzer (Analox Instruments, Lunenburg, Massachusetts, United States).

### 2.5 Experiment 1–24‐h Sucrose Preference Test (SPT)

After 48 h of alcohol abstinence in the alcohol‐drinking group, all animals underwent SPT for 24 h. Home cage water bottles were removed and replaced with 10 mL pipette sippers, one containing a 1% sucrose solution and another with tap water. Initial fluid volume was recorded, and then sippers were read at the 12‐h and 24‐h mark. At the 12‐h mark, sipper positions were swapped to control for side preference. The sippers were removed at 24 h and replaced with the home cage water bottle. This procedure was adapted from Liu et al. (2018) but contains no fluid or food deprivation.

### 2.6 Experiment 1—Forced Swim Test (FST)

After 72 h of abstinence (starting on the same day the SPT ended), animals were subjected to the forced swim test (FST). A large reservoir of tap water was set aside 24 h before to come to room temperature. Four 2‐l beakers were filled halfway with room‐temperature water and placed on a table opposite a camcorder angled down at the surface of the water. Mice were brought into the testing room 1 h prior to acclimate. At the time of test, in a randomized order, one mouse was placed in each beaker and filmed for 6 min. Afterward, animals were placed back into their home cages with a piece of paper towel to absorb excess water. Paper towels were removed at the end of the experiment. Water in the beakers was replaced every three squads with new, room temperature water from the reservoir. An observer blind to subject number scored immobility in the last 4 min of the swim for each animal. FST procedures were adapted from previous studies that demonstrated that forced abstinence from voluntary 2BC induces negative affect, though at more protracted timepoints (Vranjkovic et al. 2018).

### 2.7 Experiment 1—Novelty Suppressed Feeding Test (NSFT)

The NSFT protocol began 4 days into abstinence. At this point, cages were changed, and all food was removed, beginning a 48‐h food deprivation period. Mice had access to one large food pellet for 2 hours 23 h into the deprivation period before the pellet was removed at hour 25. Behavioral testing, then, occurred at 6 days post‐cessation; testing also occurred in the dark part of the cycle under red illumination. Animals were brought into the testing room for 1 h to acclimate; then, one at a time in a randomized order, animals were placed in alternating corners of a large plastic box (40 cm × 40 cm × 38 cm) lined with 2 cm of standard pine bedding. One pellet of food was placed in the center of the box before each mouse entered. Mice could explore for up to 5 min or until they took a bite of the center pellet. The apparatus was not cleaned between animals since most animals finished the task in under 2 min, but a new food pellet was used for each subject. An observer blind to mouse group and sex timed this latency to first feeding with a stopwatch. After time‐out or first bite, an animal was returned to their home cage and given fresh food, ending the deprivation period. A new food pellet of comparable size was used for each mouse.

### 2.8 Experiment 2–2‐h Sucrose Preference Probe

Mice were given access to alcohol and water or water alone for 5 weeks using the same procedure as in Experiment 1. Beginning in Week 2 of the drinking period, alcohol cylinders and one of the water cylinders in the control animals were removed at lights‐on on the night before the test. This was done to ensure that all animals had BECs of zero at the time of the SPT, while minimizing loss of alcohol access by making the deprivation period during the light part of the cycle. Twelve hours later, at lights‐off, remaining water bottles were removed and replaced with two serological pipette sippers: one containing water and one containing 1% sucrose as described above in the 24‐h SPT procedure. However, intake from these sippers was only measured for 2 h before alcohol and water cylinders were reintroduced for 2BC immediately after the test. This 2‐h SPT occurred weekly on the same day each week, with the positions of the sucrose and water sippers swapped each week instead of during each test. An additional 2‐h SPT occurred 48 h after cessation of alcohol drinking to match the timing of Experiment 1. The purpose of this repeated SPT was to measure the time course of any symptoms of anhedonia in what one might consider “current drinkers:” animals that had recently had alcohol access and will soon drink again, but which are not intoxicated at the time of testing.

### 2.9 Experiment 3

Experiment 3 served as a replication of both the negative affect testing in Experiment 1 and the repeated anhedonia testing during drinking in Experiment 2. The purpose was to test for anhedonia and depression during drinking as well as several time points into early abstinence, while ensuring that we only tested NSFT and FST once per subject. Animals in this cohort had the same alcohol access procedure (5 weeks of water‐10% ethanol or water–water 2BC), the same schedule of SPT probe tests during the drinking period as Experiment 2, and the same sequence of post‐drinking depression and anxiety assays except that the 2‐h SPT version was used instead of the 24‐h version. There are some notable differences, however. Animals in Experiment 3 had extra SPT probe tests during the first week of drinking, 1 week into abstinence, and 2 weeks into abstinence, unlike those animals in Experiment 2.

The animals in Experiment 3 were counterbalanced by sex and family into three groups of 24 animals (12M, 12F) for sacrifice and tissue and blood harvesting at different timepoints for a future study. Important to the details of this study is that all three groups of animals had Weeks 1–5 of SPT probe testing. One group of 24 animals had depression testing at the same timepoints into abstinence as Experiment 1 (2‐h SPT at 48 h, FST at 72 h, and 6‐day NSFT) plus an SPT test 1 week into abstinence. Another group of 24 animals received the following abstinence testing: 2‐h SPT at 48 h, 2‐h SPT at 1 week into abstinence, FST 11 days into abstinence, NSFT 14 days into abstinence, and a final SPT test 15 days into abstinence. Thus, while animals in these last two groups had multiple abstinence SPT tests, each mouse only encountered FST or NSFT once, either in early abstinence or in the second week following the beginning of abstinence.

### 2.10 Statistics

All statistical analyses were performed using Graphpad/Prism 10 software or IBM SPSS Statistics Ver. 32. The significance level for all analyses was set at *p* = 0.05. For all experiments, ethanol intake and preference data were analyzed via two‐way repeated measures ANOVAs or two‐way repeated measures mixed‐effects ANOVAs when data points were missing due to bottle leakage. Geisser–Greenhouse corrections were applied when the assumption of sphericity was violated (*p* ≤ 0.05). Additionally, a three‐way repeated measures ANOVA with median replacement was performed between Experiments 1–3 alcohol preference and intake to identify differences across experiments. The alcohol intake and preferences during the drinking rhythms experiment were analyzed via two‐way repeated measures ANOVAs. Also in Experiment 1, sucrose preference and intake in the 24‐h SPT were analyzed via two‐way ANOVA across sex and drinking history. For FST and NSFT, three‐way ANOVAs were performed across sex, drinking history, and experiment/cohort. For Experiment 3 protracted FST and NSFT, two‐way ANOVAs were run across factors of sex and drinking history.

In Experiments 2 and 3, animals had multiple 2‐h SPT probes throughout the drinking period, but because Experiment 3 had more timepoints and because some animals were sacrificed at different timepoints across Experiment 3, repeated measures analysis was only possible for a truncated section of the drinking period. For Week 1 SPT, which only existed in Experiment 3, two‐way ANOVAs with factors of sex and history were used for sucrose intake and preference. For Weeks 2–5 SPT, four‐way ANOVAs with factors of sex, history, cohort, and week were performed on both intake and preference. Two‐way repeated measures mixed‐effects ANOVAs with the Geisser–Greenhouse correction were performed on the Weeks 2–5 sucrose intake and preference with factors of history and week, and Tukey's post hoc multiple comparisons were run when applicable interactions occurred. For the 48 h, 1 week, and 2 weeks abstinence SPT tests, two‐way ANOVAs were performed on sex and history separately for each timepoint since the number of animals differed between each timepoint due to the design of the Experiment 3 replication.

In order to understand the relationships between alcohol drinking and negative affect, correlational analyses with sex as a factor were run between ethanol‐drinking variables (the average of the last three intake and preference measures) and SPT, NSFT, and FST variables. These analyses were also run between negative affective outcomes, and when 2‐h SPT test variables were used, they were the averages of the drinking period test dates (Weeks 2–5 for Experiments 2 and 3). If a sex interaction was significant, it is reported and the individual correlations by sex are reported. Otherwise, correlations are examined sex‐collapsed. In total, 31 correlations were performed (14 in Experiments 1 and 2 in Experiment 2, and 15 in Experiment 3). To avoid type I error in Experiments 1 and 3 due to the large number of comparisons, an FDR alpha correction set to alpha of 0.05 was applied using the Benjamini‐Hochberg procedure.

In Experiments 2 and 3, three‐way repeated measures ANOVAs were applied to body weight with factors of sex, history, and timepoint to ensure that body weights were the same across history groups.

## 3 Results

### 3.1 cHAPxHDID Selection

**FIGURE 2:**
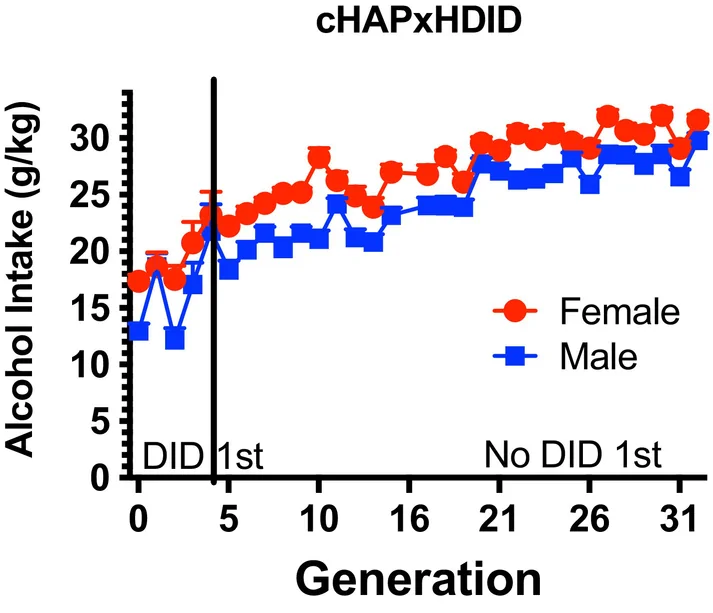
Selection response curve. Alcohol intake over generations of the cHAPxHDID line. Females tend to drink more than males (*p* < 0.0001).

Selection was generally successful at increasing daily alcohol intake in both sexes (Figure 2). Data from the first reciprocal cross of the two progenitors showed intakes at about 15 g/kg per day, consistent with additive genetics for alcohol preference, as the mean was roughly midway between that for S27 cHAP mice (about 29.5 g/kg per day), and the HDID‐1 line, which drank about 3 g/kg per day at both 9% and 12% concentrations (Crabbe et al. 2011). Selection appears to have plateaued at around 30 g/kg per day, ending up somewhat higher than the cHAP progenitor line. We had hoped that the selection limit for this line would be higher than cHAP because the HDID mice have a somewhat different progenitor (HS/Npt) than the cHAP mice (HS/lbg). In principle, mixing the alleles from two different progenitors should allow greater allelic variance, and therefore a greater variety of trait‐relevant alleles. Such outbreeding is helpful for increasing the extent of selection response. Notably, the higher intakes as compared to the cHAP line (Oberlin et al. 2011) are reflected in higher blood ethanol concentrations (see Experiment 1, below).

### 3.2 All Experiments—Two‐Bottle Choice Alcohol Intake and Preference

**FIGURE 3:**
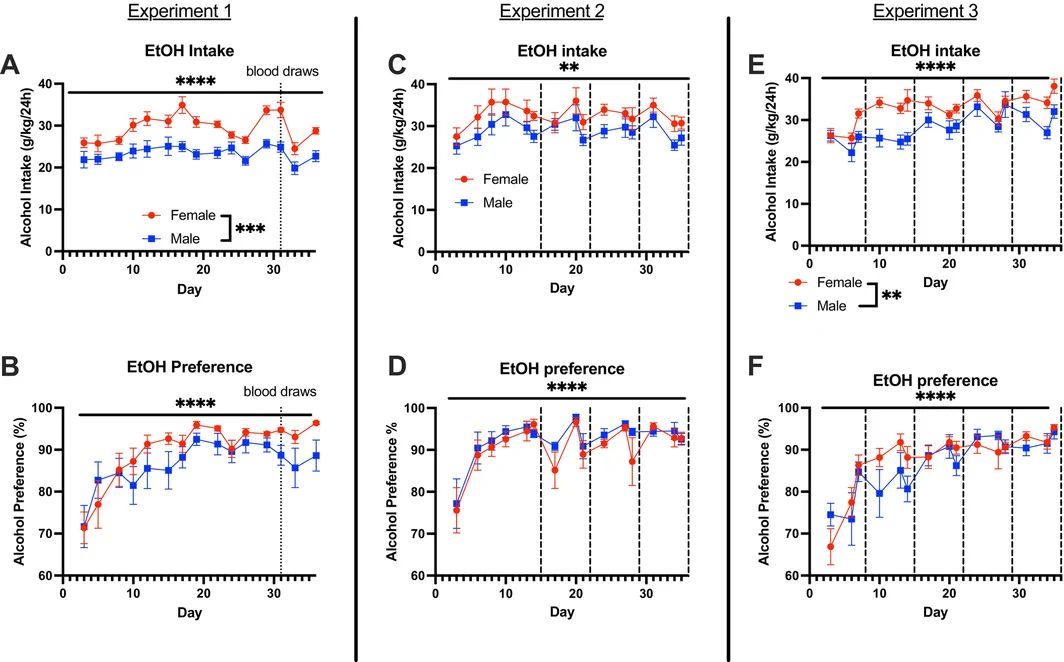
Ethanol intake and preference for all three experiments. (A) Experiment 1 ethanol intake. Intakes were high as predicted from the selection plot above. Intake changed over time (*p* < 0.0001) and females drank more than males (*p* = 0.0004). (B) Ethanol preference in Experiment 1. Preference for the 10% alcohol solution increased over time (*p* < 0.0001), but there was no sex difference. (C) Ethanol intake in Experiment 2. There was no significant main effect of sex, but intake fluctuated over days (*p* < 0.01). (D) Ethanol preference in Experiment 2. Preference between sexes did not differ, but it did increase over time (*p* < 0.0001). (E) Ethanol intake in Experiment 3. Female animals drank more than male animals (*p* < 0.01). Intake also fluctuated over time (*p* < 0.0001). (F) Ethanol preference in Experiment 3. Preference did not differ between sexes, but it did increase over time (*p* < 0.0001).

To begin, three‐way repeated measures ANOVAs were performed across experiments comparing their alcohol drinking and preference, though differences were apparent due to bottle removals in Experiments 2 and 3 a day earlier than readings occurred in Experiment 1. For intake, there was a significant main effect of time (*F*[9.196, 643.729] = 11.849, *p* < 0.001), a significant time‐by‐sex interaction (*F*[18.392, 643.729] = 5.109, *p* < 0.001), and a significant effect of experiment (*F*[2, 70] = 6.559, *p* = 0.002) and sex (*F*[1, 70] = 20.205, *p* < 0.001) indicating animals drank less in Experiment 1 and females drank more than males on average. For preference, there was a significant main effect of time (*F*[6.414, 448.98] = 30.153, *p* < 0.001) and a significant time‐by‐sex interaction (*F*[12.828, 448.98] = 2.305, *p* = 0.006) but no significant between‐subjects effects (*p*'s > 0.05). To visualize drinking in the least confusing way possible, especially with SPT manipulations included in Experiments 2 and 3, data are split by experiment and sex in Figure 3, and two‐way ANOVAs were performed for each panel.

A two‐way (sex‐by‐time) repeated measures ANOVA of the alcohol drinking animals' intake during 2BC revealed a difference over time (*F*[6.040, 114.8] = 10.24, *p* < 0.0001) and between the sexes (*F*[1, 19] = 18.41, *p* = 0.0004) with the females outdrinking the male animals, which is unsurprising in mice (Oberlin et al. 2011; Yoneyama et al. 2008). There was also a significant sex‐by‐time interaction (*F*[14, 266] = 1.859, *p* = 0.0310). Preference for the alcohol bottle also changed over time in these animals (*F*[4.213, 80.04] = 13.59, *p* < 0.0001), increasing from around 70% to 90%. There was, however, no sex difference in alcohol preference or sex‐by‐time interaction (*p*'s > 0.05). Intake and preference over time are displayed in Figure 3A,B.

The alcohol‐drinking data of Experiments 2 and 3 are analyzed separately here because of the disparity in their SPT timelines. In Experiment 2, a two‐way (sex‐by‐time) repeated measures mixed‐effects ANOVA of the alcohol intake in those animals receiving alcohol 2BC revealed that animals drank differently over days (*F*[5.800, 97.77] = 4.080, *p* = 0.001), but there was no sex or sex interaction (*p*'s > 0.05). The same analysis on alcohol preference in these animals revealed the same effects: preference changed over time (*F*[4.294, 72.38] = 8.119, *p* < 0.0001) but did not differ between the sexes, and there was no interaction (*p*'s > 0.05). Intake and preference data from Experiment 2 are visualized in Figure 3C,D, with the same from Experiment 3 shown in Figure 3E,F. In Experiment 3, a two‐way (sex‐by‐time) repeated measures mixed‐effects ANOVA of alcohol intake shows that animals drank differently over days (*F*[7.445, 252.1] = 12.52, *p* < 0.0001) and females outdrank males (*F*[1, 34] = 7.564, *p* = 0.0095). There was also a significant sex‐by‐day interaction ([7.445, 252.1] = 2.573, *p* = 0.012). The same analysis on alcohol preference in Experiment 3 indicates that preference increased over time (*F*[5.575, 188.8] = 18.29, *p* < 0.0001) but there was no main effect of sex or significant sex interaction (*p*'s > 0.05). Compared to Experiment 1, which used the same drinking period of 5 weeks, the pattern of drinking in Experiments 2 and 3 was more irregular, likely because bottles were being removed and replaced for the SPT probe tests every week. Regardless, cHAPxHDID males and females are drinking much more in the home cage here compared to B6 counterparts over similar drinking periods.

### 3.3 Experiment 1—cHAPxHDID Drinking Rhythms & BECs

A two‐way (sex‐by‐time) repeated measures ANOVA of the alcohol‐drinking animals' intake during 2BC revealed a difference over time (*F*[6.040, 114.8] = 10.24, *p* < 0.0001) and between the sexes (*F*[1, 19] = 18.41, *p* = 0.0004) with the females outdrinking the male animals, which is unsurprising in mice (Oberlin et al. 2011; Yoneyama et al. 2008). There was also a significant sex‐by‐time interaction (*F*[14, 266] = 1.859, *p* = 0.0310). Preference for the alcohol bottle also changed over time in these animals (*F*[4.213, 80.04] = 13.59, *p* < 0.0001), increasing from around 70% to 90%. There was, however, no sex difference in alcohol preference or sex‐by‐time interaction (*p*'s > 0.05). Intake and preference over time are displayed in Figure 3A,B.

**FIGURE 4:**
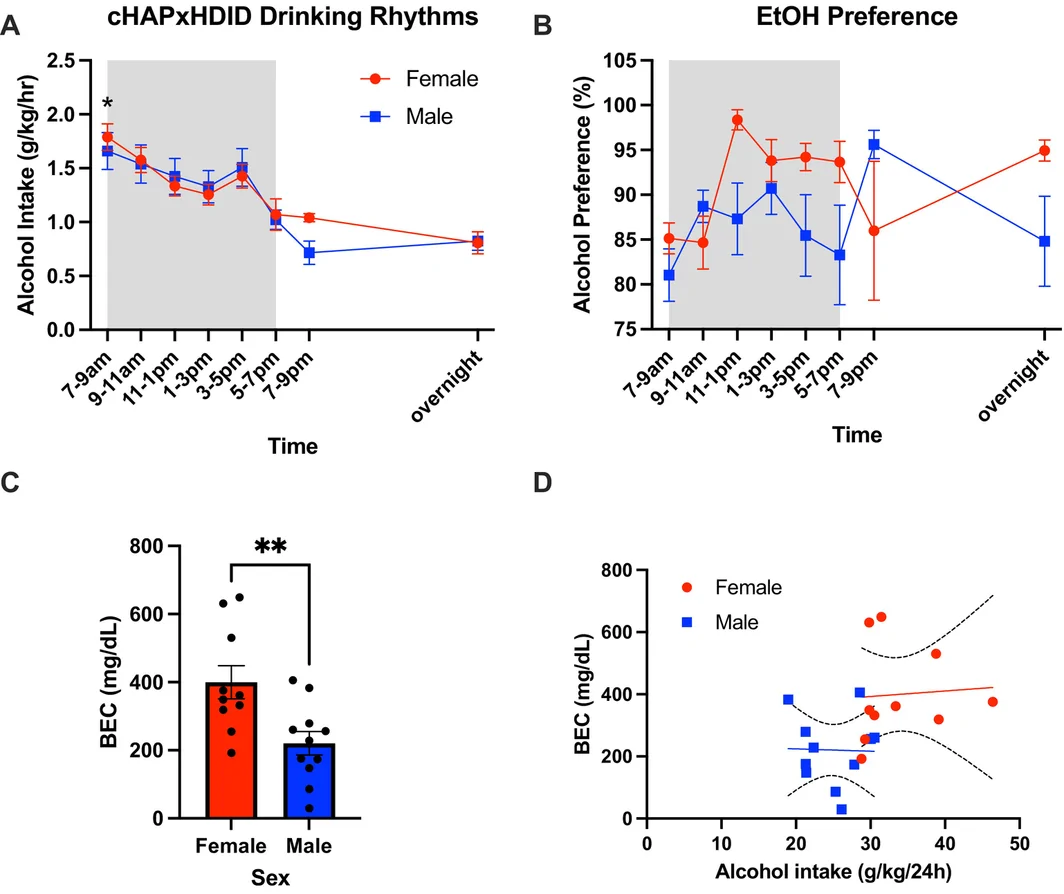
Alcohol drinking rhythms and BECs. (A) Alcohol consumption peaked early in the dark cycle (denoted by *). A two‐way repeated measures ANOVA reveals that intakes differed over time (*p* < 0.0001). Peak intake is significantly higher than all intakes recorded past 5 pm (Tukey's; all *p*'s < 0.001), indicating that intake decreases from lights‐off to lights‐on. Strikingly, animals continue to drink 0.82 g/kg/h on average in the light cycle during their inactive period. There were no sex effects. (B) Preference for the alcohol bottle during the drinking rhythm experiment. No effect of time, sex, or interaction. (C) Blood samples taken at 4:30 pm during the last week of drinking. Female animals (avg = 399.49 mg/dL) had higher BECs than male animals (avg = 220.59 mg/dL; *p* = 0.007). (D) Correlation analysis between BECs and previous day drinking split by sex. The correlation was not significant for either sex or combined across sex. There was also no significant difference between the slopes or intercepts between the sexes.

The 24‐h alcohol‐drinking pattern is displayed in Figure 4A. It generally shows animals drinking at the highest rate early in the dark part of the cycle, declining over the course of the night, and continuing at a relatively low but pharmacologically meaningful rate during the light part of the cycle. A two‐way (sex‐by‐time) repeated measures ANOVA of intake revealed a difference over 2‐h time bins (*F*[4.187, 79.55] = 11.98, *p* < 0.0001), but no sex difference and no interaction (*p*'s > 0.05). Tukey's post hoc tests revealed that the highest intake point at 7–9 am was significantly higher than the 5–7 pm, 7–9 pm, and overnight timepoints. Two local maximums in the drinking pattern appear to be at lights‐on (7 am to 9 am) and a late afternoon timepoint (3–5 pm). However, no one reading is significantly higher than every other reading, indicating a steadier drinking pattern than the cHAP animals from Matson et al. (2013) despite reaching a similar level of intake at lights‐on. Preference for the alcohol sipper is shown in Figure 4B. There was no effect of sex on preference (*p* > 0.05). Preference did not differ significantly across time bins (*p* > 0.05).

In the final week of drinking, retro‐orbital blood samples were acquired from all animals at 2 pm based on the results from Figure 4A. Only alcohol‐drinkers' blood samples were analyzed for BECs, and the results are displayed in Figure 4C,D. The cHAPxHDID mice achieved an average BEC of 305.78 mg/dL, with females (averaging 399.49 mg/dL) reaching higher BECs than male mice (averaging 220.59 mg/dL; *t* [19] = 3.047, *p* = 0.0066). BECs did not correlate significantly with the previous 24‐h intake in either sex (*p*'s > 0.05), and the slopes of these lines were not different from each other (because both were not different from zero; *p* > 0.05).

### 3.4 All Experiments—Sucrose Preference Testing

**FIGURE 5:**
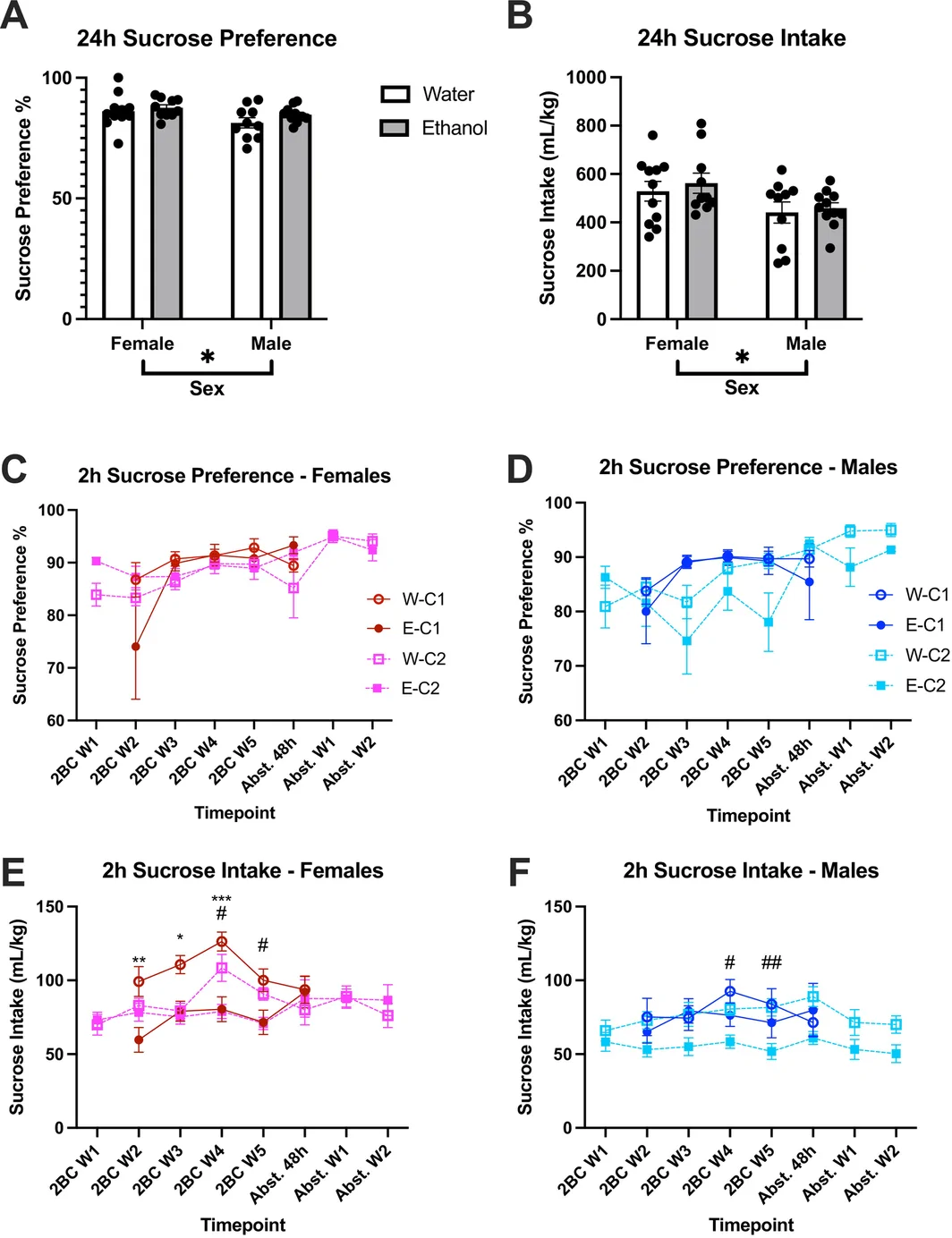
Sucrose preference tests. (A) There was no effect of drinking history on 24‐h sucrose preference, though females preferred 1% sucrose more than males (*p* = 0.03). (B) Female cHAPxHDID mice also drank more sucrose than male mice regardless of drinking history. (C, D) 2‐h sucrose preference during weeks in both Experiment 2 and 3 in Weeks 1–5 and into abstinence in females (C) and in males (D). In Week 1, alcohol‐drinking mice preferred sucrose more than water‐drinking mice, regardless of sex, though multiple comparisons are not significant. From Weeks 2 to 5, preference differed by week but did not differ between history groups. Preference also did not differ between history groups in abstinence. (E, F) 2‐h sucrose intake in both Experiments 2 and 3 in Weeks 1–5 and into abstinence in females (E) and males (F). Sucrose intake was reduced in ethanol‐drinking females in Experiment 2 during Weeks 2, 3, and 4 (*) and in Experiment 3 during Weeks 4 and 5 (#). Sucrose intake was reduced in ethanol‐drinking males in Experiment 3 during Weeks 4 and 5 (#). In abstinence, sucrose intake did not differ between history groups in either sex at any timepoint (Experiment 2: *p < 0.05, **p < 0.01, ***p < 0.001; Experiment 3: #*p* < 0.05, ##*p* < 0.01).

Affective disturbance in Experiment 1 testing began with a 24‐h‐long SPT 48 h after the cessation of drinking. Figure 5A,B show the results of this test. There was no effect of drinking history on sucrose preference or intake, and no sex‐by‐history interactions (*p*'s > 0.05). Female mice did prefer sucrose over water (*F*[1, 38] = 5.084, *p* = 0.03) and drink more sucrose (*F*[1, 38] = 6.416, *p* = 0.016) than male mice, possibly indicating a propensity for female cHAPxHDIDs to consume sweet substances.

The results of the repeated 2‐h SPT probe from Experiments 2 and 3 are shown in Figure 5C–F. In these plots, data are shown from both experiments, but analyses differed based on timepoint. Experiment 3 only included a 2‐h SPT during Week 1 of drinking. Sucrose and intake here were assessed via two‐way sex‐by‐history ANOVAs. There were no main effects or interactions on sucrose intake (*p*'s > 0.05); however, there was a main effect of history on sucrose preference (*F*[1, 67] = 5.382, *p* = 0.023), indicating that alcohol‐drinking animals preferred sucrose to water more than water‐drinking animals in this Week 1 test.

Animals in Experiments 2 and 3 all had 2‐h SPTs during Weeks 2–5. A four‐way repeated measures ANOVA (with Greenhouse–Geisser correction) was run on sucrose preference (5C and 5D), revealing a main effect of week (*F*[2.54, 251.445] = 10.93, *p* < 0.001), a week‐by‐experiment interaction (*F*[2.54, 251.445] = 5.438, *p* = 0.002), and a week‐by‐experiment‐by‐history interaction (*F*[2.54, 251.445] = 3.877, *p* = 0.014). However, there were no between‐subject main effects or interactions (*p*'s > 0.05). Overall, these results mostly indicated that preference fluctuated differently week‐to‐week between experiments dependent on history, but not significantly enough to change any interpretation of the results. A four‐way repeated measures ANOVA with factors of experiment, sex, history, and week revealed a main effect of week (*F*[3, 297] = 12.006, *p* < 0.001) and a significant week‐by‐history interaction (*F*[3, 297] = 2.785, *p* = 0.041). There were also main effects of experiment (*F*[1, 99] = 6.486, *p* = 0.012), sex (*F*[1, 99] = 11.569, *p* < 0.001), and history (*F*[1, 99] = 22.448, *p* < 0.001), and a three‐way interaction of experiment, sex, and history (*F*[1, 99] = 6.263, *p* = 0.014). Overall, these results confirm that (1) animals drank 1% sucrose differently week to week, dependent on history, (2) females drank more sucrose than males, (3) ethanol animals drink less sucrose than water animals, and (4) animals in Experiment 3 drank less sucrose than in Experiment 2. The three‐way interaction specifically was driven by the water females in Experiment 3 who tended to drink less sucrose than their equivalent counterparts in Experiment 2. Two‐way ANOVAs for each experiment were performed and can be visualized in Figure 5E,F with significance indicators for each experiment's significant pairwise comparisons within each sex.

The SPT tests occurring in abstinence are displayed in the same figures (5C–F) to illustrate changes over time more easily, but since the number of animals differed from test to test because animals were being sacrificed at different timepoints, we have analyzed sucrose intake and preference with two‐way ANOVAs using factors of sex and history for each timepoint. In short, there was no effect of history at any timepoint in abstinence for either sucrose intake or preference (*p*'s > 0.05), replicating the lack of anhedonia observed following 48‐h abstinence in Experiment 1. There were some notable sex effects in intake with female mice drinking more sucrose at the 1‐week (*F*[1, 43] = 12.39, *p* = 0.001) and 2‐week (*F*[1, 20] = 7.245, *p* = 0.014) timepoints but no sex interactions (*p*'s > 0.05). There were no sex effects or interactions on sucrose preference (*p*'s > 0.05).

### 3.5 Experiments 1 and 3—FST And NSFT

**FIGURE 6:**
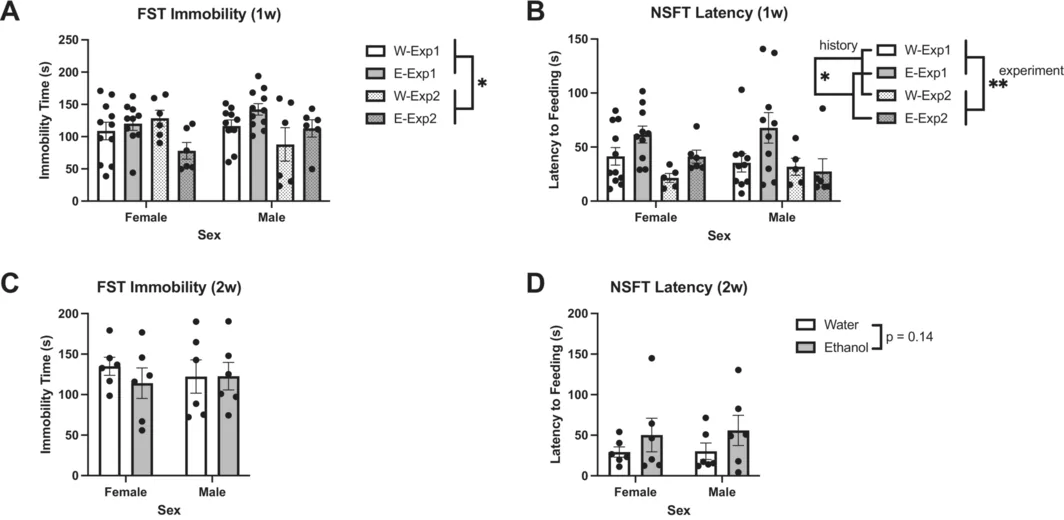
FST and NSFT at 1 and 2 weeks into abstinence. (A) Immobility in the FST during the first week of abstinence. There is a main effect of experiment (*p* = 0.04), with Exp2 animals having less immobility, and a sex‐by‐history interaction (*p* = 0.02), but no history effect (*p* > 0.05). (B) Latency to feed in the NSFT during the first week of abstinence. There is a main effect of experiment (*p* < 0.01) with animals in Exp2 taking less time to eat the pellet. There is also a main effect of history (*p* = 0.03), with alcohol history animals taking more time to eat the pellet than water history animals. (C) Immobility in the FST during the second week of abstinence. There are no significant effects. (D) Latency to feed in the NSFT during the second week of abstinence. There are no significant effects.

After 72 h of abstinence, the FST occurred in both Experiments 1 and 3. Figure 6A shows the results of the FST. A three‐way ANOVA of experiment‐by‐sex‐by‐history found an effect of experiment (*F*[1, 58] = 4.352, *p* = 0.041) and an interaction of sex by history (*F*[1, 58] = 5.467, *p* = 0.023). Tukey's post hoc tests show the only significant multiple comparison was between Experiment 3 ethanol females and Experiment 1 ethanol males.

The NSFT was administered 6 days after the cessation of drinking in Experiments 1 and 3; its temporal distance from the drinking is required by the 48 h food deprivation in the protocol. Figure 6B displays the data from the NSFT. Two animals in Experiment 3 were removed from the data because they appeared ill and lethargic at the time of testing and were subsequently sacrificed. A three‐way ANOVA of experiment‐by‐sex‐by‐history yielded a significant main effect of experiment (*F*[1, 55] = 7.896, *p* = 0.0068) and of history (*F*[1, 55] = 5.113, *p* = 0.028) and no significant interactions.

In Experiment 3 only, a subset of 24 animals had “protracted” FST and NSFT testing during the second week of abstinence. These data are shown in Figure 6C,D, respectively. There were no sex or history effects or interactions in either of these tests at the later timepoints (*p*'s > 0.05).

### 3.6 Correlational Analyses and Body Weight

Correlational analyses with sex included as a factor were performed on behavioral data in an attempt to relate drinking behavior and negative affect, and also to link negative affective behaviors together. A total of 31 correlational analyses were performed. In Experiment 1, the sex interaction term was not significant in any analysis, and neither were any of the behavioral correlations between ethanol intake and negative affective measures following alpha correction. In Experiment 2, there were no sex interactions, but 2BC intake was positively correlated with sucrose preference in the SPT probe tests in Weeks 2–5 (*r* = 0.51, *p* = 0.0255). In Experiment 3, there was a significant interaction with sex on the relationship between sucrose preference in the SPT probe tests in Weeks 2–5 and FST immobility in Week 2 of abstinence (*F*(1, 8) = 5.395, *p* = 0.0487). In males, this relationship is nonsignificant (*r* = 0.27, *p* > 0.05) but it is significant and positive in females (*r* = 0.87, *p* = 0.0257) with a steep slope. No other correlations in Experiment 3 had a significant sex interaction term or were significant when collapsed on sex following alpha correction.

In order to confirm that body weight is not driving SPT difference in Experiments 2 and 3 in this new line, three‐way ANOVAs were performed on body weight for both experiments. In Experiment 2, body weight increased over time (*F*[3, 102] = 71.64, *p* < 0.0001) and was greater in males (*F*[1, 34] = 39.47, *p* < 0.0001) with no significant history effect (*p* > 0.05) but an interaction between time and history (*F*[3, 102] = 5.583, *p* = 0.0014). In Experiment 3, body weight increased over time (*F*[1.779, 121.0] = 93.99, *p* < 0.0001) and was greater in males (*F*[1, 68] = 76.55, *p* < 0.0001) and in ethanol‐history animals (*F*[1, 68] = 8.074, *p* = 0.0059). This seems to be driven by females, though the sex‐by‐history interaction is not quite significant (*p* = 0.0587).

## 4 Discussion

### 4.1 Defining the cHAPxHDID Line

Here we describe the selective breeding and alcohol consumption, as well as begin demonstrating changes in hedonic homeostasis related to drinking of a novel line of mice, the cHAPxHDIDs, that drink much more alcohol in the home cage than existing mouse populations. This builds on previous work in our lab in deriving the cHAP mice, which reach high BECs of about 250 mg/dL in the home cage compared to B6 mice that reach ~50 mg/dL under the same conditions (Matson et al. 2013; Matson and Grahame 2013). The somewhat higher intakes and BECs observed in cHAPxHDID as compared to cHAPs are consistent with the idea that the greater the outbreeding in a progenitor, the higher the selection limit, because outbreeding avails these populations greater allelic diversity to draw upon when sculpting the final phenotype. That said, like the cHAPs, the cHAPxHDID was also constructed by crossing progenitors already enriched for high‐drinking alleles. We would further note the importance of selecting for high two‐bottle choice intake, rather than high DID BECs, in attaining these kinds of intakes even when water is concurrently available. The HDID progenitor, as stated before, drinks little to no more alcohol when water is available than its progenitor line (Crabbe et al. 2011), suggesting that when selection occurs in the absence of water, animals do not come to prefer ethanol to other fluids, only becoming intoxicated in the absence of any choice of what to drink.

The fact that cHAPxHDID mice maintain such high BECs and intakes during their active period over many weeks of drinking may be a boon to translational research because they more closely reflect the patterns of drinking and sustained, elevated BECs seen in human patients with severe AUD (Hoonpongsimanont et al. 2021; Mello and Mendelson 1970). In addition, because the cHAPs and the cHAPxHDID mice reach intense BECs and alcohol intakes, they may also be prime models for the study of the development of tolerance to alcohol's many effects; needless to say, some of the BECs observed in the cHAPxHDID mice would likely be lethal if generated by injecting a naïve mouse (Malcolm et al. 1985), so the understanding of how these mice come to be able to drink so heavily may help in understanding mechanisms and effects of tolerance in clinical AUD. We would note that none of the animals that we sampled showed a loss of righting reflex at the time their blood samples were taken, so not only were they not deceased, but they were also walking and running at these BECs. Such behavior at these BECs is not unprecedented; although we cannot yet test our mice for their driving ability, we would note that in the real world, humans do things like drive at BECs as high as 545 mg/dL (Jones 1999). Our lab has previously explored how tolerance develops in the cHAPs (Ardinger et al. 2021; Winkler and Grahame 2024), but is currently exploring the development of chronic metabolic and behavioral tolerance in the cHAPxHDIDs. We would also expect these mice to encounter significant nutritional deficits, primarily thiamine depletion, as is the case with humans who drink to these levels, and was observed in the lower‐drinking cHAP mice (Xu et al. 2021, 2019), meaning that cHAPxHDID mice likely can serve as a model of some of the toxicological sequelae of excessive alcohol consumption.

### 4.2 Negative Affect in Mice

One major advantage of using B6 mice over these selected lines for modeling effects of drinking and subsequent abstinence is their moderately consistent development of protracted negative affect following continuous access to alcohol—deficits in the FST, NSFT, and SPT 2–3 weeks following cessation of drinking (Bloch et al. 2022; Holleran et al. 2016; Vranjkovic et al. 2018), which are useful for modeling depression and anhedonia symptoms during abstinence in humans with AUD. In cHAPs, the opposite has been found at the 1‐week delay; that is, an increase in sucrose preference in alcohol history mice (Winkler and Grahame 2024). Here, we set out to see if the cHAPxHDIDs exhibited depressive or anxiety‐like behaviors, albeit at earlier timepoints more relevant to the human experience of emotional distress following abrupt cessation of drinking (see Winkler and Grahame 2025 for a translational review). While we did not find an effect of a drinking history on SPT or a significant effect of FST, latency in the NSFT at 6 days post‐drinking was elevated in mice with an alcohol‐drinking history. While the NSFT measures anxiety because it is partially an open field test, the inclusion of starvation and the food pellet in the center adds an element of motivation that taps into volitional symptoms of depression, ultimately making this a conflict assay (Blasco‐Serra et al. 2017; Samuels and Hen 2011). The current NSFT result and, to a lesser extent, the nonsignificant SPT and near‐significant FST results are opposite the affective phenotype we have documented in the cHAP mice, although they are at earlier timepoints. Early onset anxiety and depression are more common in those quitting drinking alcohol than the prolonged or protracted types studied using B6 (Brown and Schuckit 1988; Mandić‐Gajić et al. 2015; Rabinowitz et al. 2023; Winkler and Grahame 2025), and they do appear in cHAPxHDIDs after drinking much more alcohol than the inbred strain. In addition, compared to other HAP lines, these animals actually show a fairly replicable negative affective profile, and retain genetic heterogeneity which B6 mice do not. Though future research may be necessary at different timepoints to ensure that this line is a good model for affective symptoms after drinking or comorbid AUD and depression, these effects at early timepoints within the first week of abstinence more closely match the human timeline (Winkler and Grahame 2025).

AUD and depression are often comorbid (Ryan et al. 2022; Worley et al. 2012), but there has been little preclinical research that attempts to understand changes in affect prior to prolonged abstinence. Most of the comorbidity research either looks after the drinking period, as with the plethora of literature in B6 mice following DID drinking, 2BC, or after ethanol vapor inhalation (Bloch et al. 2022; Heilig et al. 2010). Alternatively, other studies look at how a “depressed” animal (induced usually by stress or trauma) drinks alcohol compared to controls (Becker et al. 2011). There are, of course, caveats and confounds to studying mood disorders across species. For example, many popular models of alcohol addiction understand the process as an allostatic drift, where continued alcohol use pushes hedonic and emotional states into continuously more negative set points (Koob 2003; Koob and Le Moal 2001). Relatedly, the self‐medication hypothesis purports that consuming alcohol relieves emotional distress (Hawn et al. 2020; Khantzian 1997), and indeed, alcohol has been shown to work as an anxiolytic (Gilman et al. 2008), although withdrawal is anxiogenic (Canver et al. 2024). Under this hypothesis, an AUD patient would report less distress while under the influence of alcohol than when sober, and this has been shown to be true for participants with AUD, though concurrent depressive diagnosis did not enhance anxiolysis (King et al. 2025). In animals, many tests for negative affect are locomotive in nature, and so intoxication complicates the interpretation of the results. Other tests, like the SPT, involve drinking a solution that could interfere with alcohol drinking itself. With these human and animal confounds in mind, we decided to measure anhedonia via SPT when alcohol was available, but mice were sober. Anhedonia seems to be a key marker for AUD/SUD and depression comorbidity prognosis in humans (Destoop et al. 2019; Garfield et al. 2014), and the SPT is a repeatable test, unlike many of the tests for other aspects of depressive‐like behavior. We also ran the test 12 h after the removal of alcohol from the cage to guarantee that animals were not intoxicated at test time. There is an argument that this could be counted as a test of depression following drinking, but humans who suffer from AUD cycle between bouts of heavy drinking and the subsequent withdrawal from these bouts during which time the highest distress is reported (Koob and Volkow 2010). The space between these binges is not counted as “remission” in humans, so we consider this interim period of emotional distress or withdrawal as contained within the drinking period. Also, it is likely during these distressing withdrawal periods that people suffering from AUD may seek help from a treatment center and self‐report the highest levels of anhedonia, depression, and anxiety. Regardless of sex, we found that alcohol‐drinking animals begin to prefer sucrose at the start of the drinking period in Week 1 with similar levels of intake to water‐drinking animals. Sucrose intake drops significantly below control levels starting in the second week of drinking and this effect persists for at least 5 weeks of alcohol availability. We interpret this difference as a hedonic disruption during the drinking period, congruent with human reports of anhedonia. The effect seemed to dissipate by 48 h into abstinence, which replicates our 24‐h‐long (starting 48‐h into abstinence) SPT results from Experiment 1. As we showed in our replication Experiment 3, where we added extra timepoints, this effect on intake did not reemerge at 1 or 2 weeks into abstinence. It should be mentioned that sucrose preference did not differ between alcohol and control animals at any point past Week 1 during drinking or in abstinence. In future studies, we aim to understand the molecular and neuronal source(s) of this potential marker of anhedonia. We are particularly interested in the intersection of neuroinflammation, thiamine deficiency, and depressive symptoms, as the cHAPs, one of the cHAPxHDID parent lines, have shown consistent inflammation and thiamine deficiency after 7 months of continuous alcohol access (Xu et al. 2021). There also may be a connection between this anhedonia phenotype and craving—there is a plethora of literature expounding the process by which individuals with AUD prefer drug reward over natural reward (Anselme 2009; Nall et al. 2021), and it may be that craving and anhedonia are two sides of the same coin, and thus, could be manipulated via shared neurocircuitry. Combining the results of all of the experiments, a reduction in sucrose intake in alcohol‐drinking animals may be the result of removal of alcohol during the light period during which cHAPxHDIDs still drink pharmacologically relevant levels of alcohol when they should be sleeping, as evidenced in Figure 4A. Without alcohol access overnight, reintroduction of two fluids to the cage that do not contain the desired drug reward may manifest as a disinterest to consume the same level of sucrose as water‐drinking animals.

### 4.3 Limitations

While an alcohol history consistently lowered sucrose intake in Experiments 2 and 3, we did not find a history effect in Experiment 1. There could be many reasons for this discrepancy: 24‐h vs. 2‐h sucrose access time, one‐off testing vs. repeated testing, and testing in abstinence vs. during the drinking period. Since our goal was to characterize changes in hedonic value of sucrose during the drinking period, it is not completely shocking that the 24‐h measure at 48‐h into abstinence differs from our probe tests. In fact, the same 48‐h timepoint in Experiments 2 and 3 also yielded a null result in the 2‐h version of the SPT, suggesting that the anhedonic effect of alcohol consumption wanes rapidly during abstinence.

We also found a consistent sex effect in alcohol drinking but only some sex‐specific effects in our affective testing. In Experiment 2, the SPT intake effect was only found in female mice even though female mice actually did not have significantly higher intake in 2BC, and when animals do show sex disparity in alcohol intake in Experiments 1 and 3, they don't show differences in negative affect in NSFT or FST, save for an increase in females in 24‐h SPT metrics regardless of history in Experiment 1. In that way, while they drink more than inbred strains, the cHAPxHDID animals may resemble B6 mice in that differences in baseline locomotion, consummatory behavior, and type of alcohol administration paradigm can change the outcomes of negative affective behavioral assays (Salazar and Centanni 2024). Future studies could explore other facets of anxiety and depression, such as those that may affect one sex more than another (e.g., social interaction). Additionally, we found little evidence that alcohol intake was correlated with negative affective measures or that signs of negative affect were correlated with each other. In Experiment 2, animals that drank more alcohol in 2BC tended to prefer sucrose to a higher degree. This could be because the animals who drink more are better at tracking the sipper that contains the non‐water substance, but then again, alcohol animals did not prefer the sucrose solution more than water animals. In Experiment 3, sucrose preference was associated with more immobility in the FST during Week 2 of abstinence testing. This relationship is difficult to explain, but we think it may be due to exceptionally low variability in sucrose preference (range of 90%–94%) in that group of six female animals compared to the males. The group size in the FST and NSFT was much reduced compared to the other experiments due to the sacrifice of animals at different timepoints, so examining this relationship with more animals is another future direction.

Finally, while body weight did not obfuscate the results of Experiment 2, it did differ between the ethanol and water groups in Experiment 3, with ethanol animals weighing more than water animals, especially in the female animals. Thankfully though, the effect we find of lowered intake in SPT in ethanol‐history animals is still quite robust in groups that do not differ in body weight (Experiment 2 females and Experiment 3 males). In fact, the Experiment 3 female animals were slow to replicate Experiment 2 until Week 4 despite the difference in body weight. Counterbalancing occurred by sex and by family (parents of the subjects) before alcohol was given to any mice, but weight must be considered for future replications of this paradigm.

## 5 Conclusion

Taken together, these data suggest that, like their cHAP predecessors, cHAPxHDID mice drink readily in the home cage and have intakes and BECs that far exceed B6 mice. Unlike cHAPs, however, these animals display high drinking during the inactive period and some negative affect following drinking, significantly in the NSFT 6 days into abstinence. In an attempt to model concurrent depression and AUD, we also ran an SPT probe experiment whereby cHAPxHDID mice displayed reduced sucrose intake from the second week onward during 2BC and then normalized to control levels at 48 h into abstinence. This reduction in sucrose intake was specific to during‐drinking and did not return 1 or 2 weeks after cessation of 2BC. In future studies with these animals, we intend to explore this behavioral effect, along with investigating affect at other timepoints following drinking to ensure that cHAPxHDID mice are good candidates for preclinical AUD + MDD comorbidity research. Additionally, respecting half of their namesake, we intend to explore the cHAPxHDID's binge drinking patterns in drinking‐in‐the‐dark and compare them with other selectively bred lines.

## Funding

This work was supported by the National Institute on Alcohol Abuse and Alcoholism, AA07611, AA007462.

## Conflicts of Interest

The authors declare no conflicts of interest.

## Data Availability

The data that support the findings of this study are available from the corresponding author upon reasonable request.
